# Mixed Bullous-Eczematous Contact Dermatitis From a Black Henna Tattoo in an African American Female With Sickle Cell Disease With Post-Dermatitis Pain

**DOI:** 10.7759/cureus.9200

**Published:** 2020-07-15

**Authors:** Dharam Persaud-Sharma, Marien Govea, Robert Hernandez

**Affiliations:** 1 Internal Medicine, Kendall Regional Medical Center/Herbert Wertheim College of Medicine, Florida International University, Miami, USA; 2 Internal Medicine, Herbert Wertheim College of Medicine, Florida International University, Miami, USA; 3 Internal Medicine/Infectious Disease, Kendall Regional Medical Center, Miami, USA

**Keywords:** sickle cell disease, henna tattoo, hypersensitivity reaction, inflammation, burn, black henna tattoo

## Abstract

Traditionally practiced in East Asian and Southeast Asian countries, Henna tattooing has gained western popularity in creating temporary decorative patterns on the skin. Derived from the Lawsonia inermis shrub prevalent in Asia/Southeast Asia, the leaves of this plant are ground to create a paste with a brown pigment commonly called Mehndi or Henna which have deep-rooted cultural values/practices. The pure organic form of these compounds has few reported side effects. However, with gaining western popularity, synthetic additives to the natural paste to create color variation, shorten application times, and increase shelf-life have led to an increase in the incidence of adverse reactions. Namely attributed to synthetic compounds like para-phenylenediamine (PPD) or para-toluylenediamine, this synthetic type of mixture is called black henna. Although multiple types of adverse reactions with black henna have been documented as an eczematous type of reaction, few if any cases of adverse reactions of black henna affecting patients with sickle cell disease (SCD) have been documented. In this case, we aim to present an atypical mixed bullous-eczematous contact dermatitis reaction secondary to a PPD containing black henna dye applied to the skin of a patient with homozygous SCD. We intend to raise awareness of the deleterious cosmetic sequelae and chronic post-dermatitis pain manifestations which may arise in patients with SCD, as the popularity of black henna tattooing grows in the United States where SCD is one of the most prevalent hemoglobinopathies amongst black Americans.

## Introduction

According to the Center for Disease Control and Prevention (CDC), nearly 100,000 Americans are affected by sickle cell disease (SCD). A closer look indicates that one out of every 365 African American births are diagnosed with the disease compared to one out of every 16,300 Hispanic-American births [1]. SCD is an autosomal recessive inherited mutation to the beta-globin gene which is characterized by a Glu6Val point mutation, where valine replaces glutamic acid. In SCD, the oxygen carrying hemoglobin molecules are atypically crescent shaped from their normal oval bodies. This causes two effects: the first is a decreased oxygen carrying capacity and the second is a propensity for vaso-occlusion in blood vessels which could be fatal in patients with SCD. Symptomatically, SCD patients often develop ischemic pain crises due to the blockage of blood vessels by low oxygen carrying crescent-shaped cells limiting oxygen delivery to distal region of extremities. This is formally called the hand-foot syndrome, which causes swelling to the bilateral extremities. Other well-known complications that may arise can range from chronic musculoskeletal pain, swelling of the digits of the hands and feet which is called dactylitis, delayed wound healing due to impaired angiogenesis affecting blood products and lymphocytes coupled with a reduced release of endothelial progenitor cells from bone marrow, and autosplenectomy due to infarctions of the spleen secondarily to splenic artery occlusions [2,3]. This often leads to immunocompromising and susceptibility to encapsulated organisms like *Streptococcus pneumoniae, Haemophilus influenzae, and Neisseria meningitidis, *which are usually protected by the spleen.

Henna, also known as Mehndi, is produced by grinding leaves from the Lawsonia inermis plant to create temporary staining of the skin. The use of this application pre-dates 5,000-year civilizations in North Africa, the Middle East, and India where it is still used for religious and cultural ceremonies [4]. The staining element of Henna, called hennotanic acid, will penetrate depths as deep to the stratum granulosum, which will eventually evolve to become the stratum corneum creating the transient nature of the skin stain. To increase shelf life, enhanced stain duration, shorten application times, and the creation of varied colors, synthetic chemicals like para-phenylenadiamine (PPD) or para-toluylenediamine have been added to natural brown henna which have proven in some instances to cause allergic adverse skin reactions [5]. Certain populations with morbidities are more susceptible than others for these reactions, especially those with hemoglobinopathies such as SCD and glucose-6-phosphate dehydrogenase (G6PD).

In this case report, we are presenting such a case of a patient with SCD who had an atypical and exaggerated skin reaction after receiving a black henna tattoo which resulted in mixed bullous-eczematous type of dermatitis with a post-dermatitis pain reaction. 

## Case presentation

**Figure 1:**
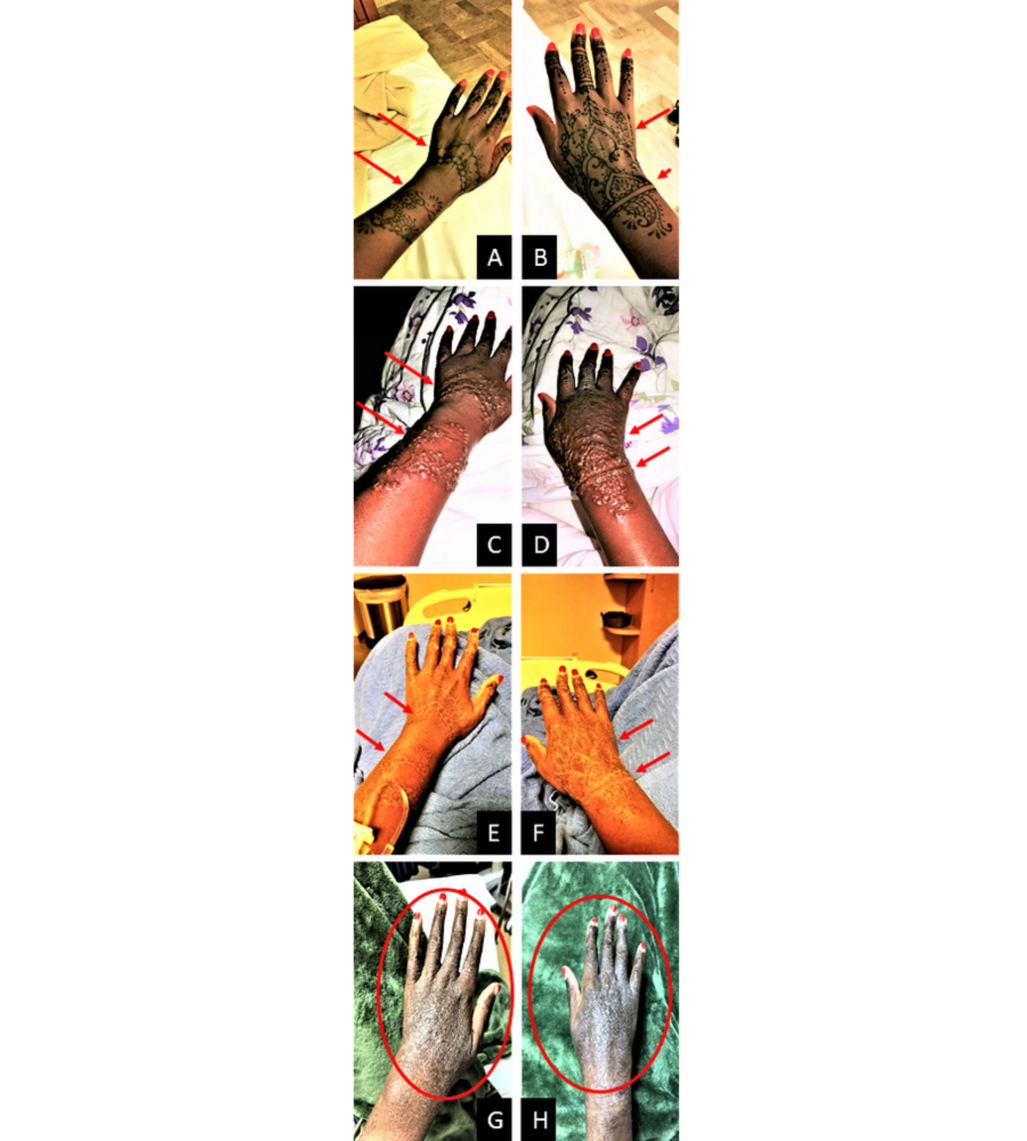
Timeline and Progression of Type IV Hypersensitivity Reaction from Black Henna Tattoo (A) Left hand with newly applied black henna tattoo. (B) Right hand with newly applied black henna tattoo. (C) Left hand bullous reaction 72 hours after black henna tattoo. (D) Right hand bullous reaction 72 hours after black henna tattoo. (E) Left hand after anti-bacterial whirlpool debridement. (F) Right hand after anti-bacterial whirlpool debridement. (G) Left hand two months post-exposure showing eczematous type reaction. (H) Right hand two months post-exposure showing eczematous type reaction.

This is a case of a 45-year-old, uninsured, African American female with a medical history of SCD with a reported blood transfusion history, who presents with chronic pain in both of her hands after suffering a mixed bullous-eczematous type IV hypersensitivity reaction secondary to a black henna tattoo. The patient’s medical history is otherwise unremarkable without a history of any skin tattoos (temporary or permanent) or hair dye use. She reports that while vacationing in Mexico, she received a black henna tattoo on the dorsum of her hands that extended to her mid-forearms bilaterally (Figures 1A, 1B). The patient reported that on the day of receiving the tattoo, she felt a “burning” sensation at the site of tattoos with “warm-hot” feeling. This progressed with a feeling of subcutaneous edema. After returning to the United States, three days after receiving the tattoo, the patient experienced a bullous type of inflammatory reaction with desquamation of the skin, which prompted her to seek emergency medical attention (Figures 1C, 1D). At that point in time, the patient also reported shortness of breath and 10/10, non-radiating “unbearable pain” in the hands. While in the emergency department (ED), it was determined that she was suffering a bullous type IV hypersensitivity reaction. Nikolsky sign was not documented at the time of the examination, and no skin biopsies were obtained which is a limitation of clinical examination to characterize the nature of the bullae. However, desquamation of both the dermis and epidermis in some regions was appreciated on clinical examination, mimicking second-degree superficial partial thickness burns as noted by evaluation of the plastic surgeon. A chest radiograph was obtained to rule out an acute chest syndrome. Her pain was managed with pharmacotherapy, and she was further treated with a chilled anti-bacterial whirlpool hydrotherapy of the hands and forearms (Figures 1E, 1F). Anterior-posterior and lateral radiographs of the hands were obtained which did not reveal any concerns for foreign bodies, bony infiltration, or clostridial myonecrosis type of infections. She received a tetanus vaccine due to her unknown vaccination status. Routine labs, including a complete blood count and complete metabolic panel, were obtained for evaluation of infection. The patient had vital signs within normal limits, was afebrile, and had unremarkable labs and chemistry. She was later discharged with a seven-day course of prophylactic antibiotics, pain control, and a dose of oral steroids.

Two months later, the patient returned to the ED with complaints of ongoing chronic pain at the site of the tattoo. Clinical examination revealed a new eczematous type IV hypersensitivity reaction on the skin (Figures 1G, 1H). Her hands were neurovascularly intact, and she had mild reduction in the range of motion of the metacarpophalangeal and wrist joints bilaterally. The patient reported that she was using soap and water as wound care since her discharge without application of any ointments or creams. In the ED, her pain was again controlled with non-opioid pharmacotherapy. Boudreaux’s Butt Paste was applied to the surface of her skin. She was later admitted to the hospital for further evaluation. She was consulted for pain management for further outpatient follow-up. She was also consulted with plastic surgery, who signed-off on the case without the need for surgical intervention and was later discharged with wound care instructions using calamine lotion.

## Discussion

Although aesthetically appealing, Henna tattooing can be an unknown problem for many patients depending on (co)-morbidities like SCD and G6PD. Synthetic chemicals, such as PPD and para-toluylenediamine amongst other similar types of toxins, are known to cause a skin sensitizing effect [5]. Prior exposure to PPD can lead to an immediate type I hypersensitivity reaction that can cause fatal asthmatic attacks, bronchospasms, as well as hives and rhinitis [6]. In addition, populations with G6PD and SCD present with severe hemolytic episodes that begin 24-72 hours after black henna application, with prolonged anemic episodes [5,7]. However, the most commonly reported reaction that follows a black henna tattoo that is not limited to patients with G6PD is a type IV hypersensitivity reaction, contact dermatitis as seen in Figure 1. PPD has proven to be problematic in patients without hemoglobinopathies, and a growing incidence of adverse reactions has been published. Some of these complications include dermatitis, pruritic eczema, ulcerations, angioedema with topical applications as compared to rhabdomyolysis, anemia, and renal failure if ingested [8,9]. One of the challenges to minimize the incidence of these dermatological reactions include regulation and monitoring of cosmetic product ingredients. Some monitoring agencies like the European Commission have listed PPD as a dangerous cosmetic product [10].

Type 4 hypersensitivity reactions can be further categorized into contact, tuberculin, and granuloma responses with varying reaction times ranging from 48 to 72 hours (contact and tuberculin type) up to 21-28 days (granulomatous type). A contact reaction, such as the application of black henna, occurs when T-cells from the stratum basale and lymphocytes and macrophages from the dermis reticularis cause edema and erythema consistent with an eczematous reaction [11]. A bullous reaction may also occur with multiple fluid-filled vesicles forming due to the concentration of T-cells in the epidermis as seen in Figure 1 [12]. Also, skin biopsies were not taken from the patient to document the expected elevated levels of T-cells in the skin due to PPD inducing a T-lymphocyte-mediated allergic response; however, expected physiology can explain clinical manifestations observed in this patient. The patient experienced a bullous type of reaction within the first 72 hours of the black henna tattoo, which of itself is rare to cause contact dermatitis. One of the limitations of this study included not performing a patch test to definitively determine the immunological etiology of the allergic contact dermatitis. However, due to the patient’s strong dermatological response after the black henna tattoo, patch testing with the same agent was not recommended. Additionally, the patient was lost for further outpatient follow-up.

It is believed that these clinical manifestations have been augmented due to the patient's prior history of blood transfusions, thereby causing erythrocytes alloimmunization due to antigenic differences between donor blood and her own blood type, increasing her immunosensitive to foreign antigens, like those found in the various layers of the skin from black henna tattoos that would otherwise be non-reactive or minimally reactive in patients without such hemoglobinopathies [12,13]. Typically, patients who experience contact dermatitis from various sources do not have long-lasting skin scarring or prolonged hypersensitivity reactions as was observed in this patient. Once the insulting surface antigen is removed from contact, recovery usually ensues with minimal cosmetic aftereffects. However, in this patient it appears that despite recommended wound care, the patient continued to develop an example of this type of reaction that was evident two months after exposure to the antigen. This can be explained by the previously documented prolonged hemolysis observed in patients with G6PD or SCD. Additionally, it has been hypothesized that a delayed and recurrent reaction occurs because the tattoo particles are embedded in the dermis and have not been removed so they resurface and aggravate a repeated episode of a delayed cellular reaction. This is starkly different from traditional contact dermatitis which results from surface irritation of allergens, as compared to embedded tattoo particles. The combination of these phenomena is believed to contribute to her development of post-dermatitis pain which has led to her readmission to the hospital for further evaluation. It is hypothesized that the pain that she was experiencing was not different than the pain experienced by SCD patients in acute pain episodes imparted by ischemia.

This matter is further complicated because of the subjective nature of pain, and the advent of the opioid epidemic, wherein a perceived over exaggeration of symptoms by patients is often encountered in the EDs in the United States [14]. However, in this specific case, it is believed that the patient developed a hypersensitivity to pain due to changes in afferent neurons in the central nervous system due to repeated pain episodes, which was previously supported by findings in animal studies [15]. Eczematous dermatitis of the patient's extremities with scarring mimicking that of second-degree burns may have physiologically changed mechanoreceptors, nociceptors, and thermoreceptors in the dermis and epidermis of her extremities. It is unknown whether these are permanent or changes that can be normalized after an extended period. 

## Conclusions

Henna tattooing is aesthetically pleasing and well enjoyed by most individuals but can be problematic in certain populations of people with morbidities such as hemoglobinopathies. This case report exemplifies such adverse reactions that can occur when patients with such conditions are exposed to antigens like synthetic chemicals PPD and para-toluylenediamine added to traditionally natural henna dye from the Lawsonia inermis plant. This is a case of a 45-year-old female with SCD, who not only experienced an atypical bullous type of contact dermatitis, believed to be due to alloimmunization from prior blood transfusions common to patients with SCD, but also experienced a prolonged wound healing mainly due to impaired angiogenesis, and increased post-dermatitis pain due to increased and prolonged hemolysis as previously documented in patients with SCD. Although without skin biopsies or confirmatory immunologic patch testing to document a skin allergy, direct visualization of these reactionary skin changes in the can explain the clinical presentation of her symptoms. This paper proposes a cautionary measure for clinical counseling of patients with hemoglobinopathies contemplating receiving a black henna tattoo and highlights the need for further clinical studies to explore the effects of PPD and hemoglobinopathies.
